# Examining Risky Driving Behavior Among Older Drivers Using Self-Reported and Objective Data

**DOI:** 10.1093/geroni/igaa057.1507

**Published:** 2020-12-16

**Authors:** Jennifer Zakrajsek, Lisa Molnar, David Eby, David LeBlanc, Lidia Kostyniuk, Nicole Zanier, Tina Sayer

**Affiliations:** 1 University of Michigan, Ann Arbor, Michigan, United States; 2 Toyota Motor North America Research & Development, Ann Arbor, Michigan, United States

## Abstract

Motor vehicle crashes represent a significant public health problem. Efforts to improve driving safety are multifaceted, focusing on vehicles, roadways, and drivers with risky driving behaviors playing integral roles in each area. As part of a study to create guidelines for developing risky driving countermeasures, 480 drivers (118 young/18-25, 183 middle-aged/35-55, 179 older/65 and older) completed online surveys measuring driving history, risky driving (frequency of engaging in distracted [using cell phone, texting, eating/drinking, grooming, reaching/interacting] and reckless/aggressive [speeding, tailgating, failing to yield right-of-way, maneuvering unsafely, rolling stops] driving behaviors), and psychosocial characteristics. A cluster analysis using frequency of the risky behaviors and seat belt use identified five risky behavior-clusters: 1) rarely/never distracted-rarely/never reckless/aggressive (n=392); 2) sometimes distracted-rarely/never reckless/aggressive (n=33); 3) sometimes distracted-sometimes reckless/aggressive (n=40); 4) often/always distracted-often/always reckless/aggressive (n=11); 5) no pattern (n=4). Older drivers were more likely in the first/lowest cluster (93.8% of older versus 84.2% of middle-aged and 59.3% of young drivers; p<.0001). Fifteen older drivers participated in a follow-up study in which their vehicles were equipped with a data acquisition system that collected objective driving and video data of all trips for three weeks. Analysis of video data from 145 older driver trips indicated that older drivers engaged in at least one distracted behavior in 115 (79.3%) trips. While preliminary, this suggests considerably more frequent engagement in distracted driving than self-reported and that older drivers should not be excluded from consideration when developing risky driving behavior countermeasures. Full study results and implications will be presented.

